# Synthesis of Silver Nanocomposite Based on Carboxymethyl Cellulose: Antibacterial, Antifungal and Anticancer Activities

**DOI:** 10.3390/polym14163352

**Published:** 2022-08-17

**Authors:** Salem S. Salem, Amr H. Hashem, Al-Aliaa M. Sallam, Ahmed S. Doghish, Abdulaziz A. Al-Askar, Amr A. Arishi, Amr M. Shehabeldine

**Affiliations:** 1 Botany and Microbiology Department, Faculty of Science, Al-Azhar University, Nasr City 11884, Cairo, Egypt; 2 Biochemistry Department, Faculty of Pharmacy, Ain-Shams University, Abassia, Cairo 11566, Cairo, Egypt; 3 Department of Biochemistry, Faculty of Pharmacy, Badr University in Cairo (BUC), Badr City 11829, Cairo, Egypt; 4 Biochemistry and Molecular Biology Department, Faculty of Pharmacy (Boys), Al-Azhar University, Nasr City 11231, Cairo, Egypt; 5 Department of Botany and Microbiology, Faculty of Science, King Saud University, Riyadh 12372, Saudi Arabia; 6 School of Molecular Sciences, The University of Western Australia, Perth, WA 6009, Australia

**Keywords:** silver nanoparticles, carboxymethyl cellulose, antimicrobial, anticancer

## Abstract

Traditional cancer treatments include surgery, radiation, and chemotherapy. According to medical sources, chemotherapy is still the primary method for curing or treating cancer today and has been a major contributor to the recent decline in cancer mortality. Nanocomposites based on polymers and metal nanoparticles have recently received the attention of researchers. In the current study, a nanocomposite was fabricated based on carboxymethyl cellulose and silver nanoparticles (CMC-AgNPs) and their antibacterial, antifungal, and anticancer activities were evaluated. The antibacterial results revealed that CMC-AgNPs have promising antibacterial activity against Gram-negative (*Klebsiella oxytoca* and *Escherichia coli*) and Gram-positive bacteria (*Bacillus cereus* and *Staphylococcus aureus*). Moreover, CMC-AgNPs exhibited antifungal activity against filamentous fungi such as *Aspergillus fumigatus*, *A. niger*, and *A. terreus.* Concerning the HepG2 hepatocellular cancer cell line, the lowest IC_50_ values (7.9 ± 0.41 µg/mL) were recorded for CMC-AgNPs, suggesting a strong cytotoxic effect on liver cancer cells. As a result, our findings suggest that the antitumor effect of these CMC-Ag nanoparticles is due to the induction of apoptosis and necrosis in hepatic cancer cells via increased caspase-8 and -9 activities and diminished levels of VEGFR-2. In conclusion, CMC-AgNPs exhibited antibacterial, antifungal, and anticancer activities, which can be used in the pharmaceutical and medical fields.

## 1. Introduction

Cancer is considered the second largest cause of death worldwide and has historically been one of the most common diseases with the highest mortality rates [1]. According to estimates, there will be roughly 10.0 million cancer deaths and 19.3 million new cases in 2020. Previously, chemotherapy was used to treat cancer without a clear understanding of the target, protein, or enzyme responsible, leading to the inhibition of the entire family of enzymes or receptors and causing high levels of toxicity and side effects. However, today’s anticancer medications are highly selectively on specific subtypes of clear targets like cyclin-dependent kinases, the Ras protein, epidermal growth factor receptors, and cancer stem cells [2,3]. Many compounds have anticancer activity, such as isoxazole–carboxamide derivatives [1], safrole oil [4], and benzoxazole [5]. Multi-drug resistant bacteria emerged due to the overuse or misuse of antibiotics; additionally, the modern treatment of antibiotics via clinically prescribed dosages is not able to manage these pathogens, and therefore, preventive strategies are necessary [6]. Likewise, fungi became more resistant to common antifungal agents; thus, pathogenic fungi invade more than 1.2 billion individuals worldwide, with at least 1.7 million deaths per year [7,8,9]. Mortality of fungal pathogens becomes equal to drug-resistant *Mycobacterium tuberculosis* and exceeds that of malaria [10]. 

Nanotechnology has the potential to revolutionize a wide array of applications in the fields of catalysis, sensors, optoelectronics, drug delivery, antimicrobial agents, vector control, and anticancer agents. Recent years have seen the rapid synthesis of nanocrystals (Ag, Zn, Se, Au, and Cu) using biological (plants, fungi, algae, and bacteria) techniques [11,12,13,14,15,16,17,18]. Integrating nanomaterials (NMs) with biodegradable polymers is the subject of current research. Polysaccharides are biocompatible, non-toxic, and quickly biodegradable, and they are plentiful. Polysaccharides are widely employed as a suitable medium for reducing and stabilizing metal nanoparticles (MNPs) due to their environmentally benign properties and ease of processing into various hydrogel forms [11,19,20]. Nanoparticles (NPs) increase biopolymers’ thermal, mechanical, and barrier characteristics, allowing them to be used in wider applications [21,22,23,24]. Because of its notably renewable, biodegradable, and non-toxic qualities, carboxymethyl cellulose (CMC) is among the most commonly used cellulose derivatives, with applications in the medical, agricultural, and environmental industries [25]. It is a water-soluble polymer, having carboxymethyl groups attached to some of the three monomers that make up the cellulose skeleton’s backbone [25,26]. CMC has been frequently used as a suitable stabilizer in producing AgNPs in an aqueous media when other compounds are utilized as reducing agents [27]. To decrease production costs and employ safe materials as CMCs for therapeutic applications, significant efforts are being undertaken to develop cost-effective and ecologically acceptable processes for Ag^+^ reduction and fabricating silver nanoparticles (Ag-NPs) [28]. AgNPs have been commonly applied as antimicrobial agents in the medical sector, used for fabric coatings and various biomedical applications after proving to be largely harmless to human cells [13,29,30]. Because of their huge specific surface area proportion of surface atoms, AgNPs have better antimicrobial capabilities than bulk silver [31,32]. Therefore, AgNPs are used in the fighting of multidrug resistance bacteria due to their biological activities [33]. The production of AgNPs for efficient cancer therapy, investigation, and diagnostics was examined by researchers [34,35]. AgNPs can be linked and/or loaded with various medicines, polysaccharides, and nanostructures to improve their sensitivity and efficacy against various tumor cells [36,37]. This study aims to prepare and characterize a new nanocomposite based on carboxymethyl cellulose and silver nanoparticles (CMC-AgNPs) and to study their antibacterial, antifungal, and anticancer activities. 

## 2. Materials and Methods

### 2.1. Chemical and Reagents Used

Taxol was obtained from Sigma Chemical (St. Louis, MO, USA). Dimethyl sulfoxide and MTT were purchased from Sigma (Sigma, St. Louis, MO, USA). DMEM, penicillin/streptomycin (Pen/Strep) solution (Pen/Strep), phosphate buffer saline (PBS), fetal bovine serum (FBS), and trypsin-EDTA were brought from Gibco (Gibco, TFS, Inc., Wake Forest, NC, USA), malt extract agar (MEA)(Oxoid, Lenexa, KS, USA).

### 2.2. Synthesis of Carboxymethyl Cellulose/Silver Nanoparticles (CMC-AgNPs)

Using a chemical reduction procedure with carboxymethyl cellulose as a reducing and stabilizing agent, carboxymethyl cellulose/silver nanoparticles (CMC-AgNPs) were created. The method described by Hebeish et al. [38] was used to synthesize carboxymethyl cellulose/silver nanoparticles (CMC-AgNPs), with a few minor modifications. Carboxymethyl cellulose (0.1%) was produced by mixing 100 mL DW. NaOH and AgNO_3_ was made from this. The Carboxymethyl Cellulose solution received an aliquot of 2 mL of 2 mM AgNO_3_ and 100 µL of 0.5 M NaOH. Carboxymethyl Cellulose solution was stirred for 4 h at 85 °C. The colorless carboxymethyl cellulose solution changed from colorless to yellow and eventually brown, demonstrating the synthesis of CMC-AgNPs. Then, The CMC-AgNPs were dried at 90 °C for 24 h. The final product was collected and stored for analysis.

### 2.3. Characterization of CMC-AgNPs

A variety of instrumental analytical methods were used to characterize the CMC-AgNPs. The total internal spectra were used to semi-quantitatively measure the visible IR spectrum of the CMC-AgNPs by measuring the transmittance over a spectral region between 4000 and 400 cm^−1^ using a spectrum, two IR Spectrometer (PerkinElmer Inc., Shelton, CT, USA.). All spectra were collected at a 4 cm^−1^ resolution. A Diano X-ray diffractometer (Philips, Amsterdam, The Netherlands) with a Cu-K source (λ = 0.15418 nm) activated at 45 kV, as well as a generator (PW,-1930) and a goniometer (PW,-1820), were used to study the XRD pattern of the produced CMC-AgNPs. The prepared CMC-AgNPs were examined using the TEM technique to determine their size and morphology. A 200 kV voltage ultra-high-resolution TEM (JEOL-2010) was used. Moreover, SEM analysis (SEM, ZEISS, EVO-MA10, Jena, Germany) was used to elucidate the surface morphology, boundary size, and the distribution of the synthesized CMC-AgNPs. To study the elemental composition, purity, simplicity, and the distribution of elements shaping the prepared CMC-AgNPs, EDX, BRUKER, Nano GmbH, D-12489, 410-M, Germany was employed. 

### 2.4. In Vitro Antibacterial Activity and MIC Determination

*Klebsiella oxytoca* ATCC 51983 & *Escherichia coli* ATCC 35218 & *Staphylococcus aureus* ATCC 25923, and *Bacillus cereus* ATTC 11778 were purchased from the American Type Culture Collection (ATCC) and were chosen for the antibacterial screening. In addition, bactericidal activity was validated utilizing known antibiotics, namely ciprofloxacin [39]. An agar-well diffusion method was applied to determine antibacterial properties [40]. Firstly, 100 µL of preserved bacteria were transferred into nutrient agar plates, distributed by streaking, and incubated at 37 ± 2 °C for 24 h [41]. Afterward, 20 μL of the tested CMC/Ag-NPs (2 mg/mL) were added to each well and left in the refrigerator for 1 h. Then, they were incubated overnight at 37 ± 2 °C. After the incubation period, the diameters of clear inhibition zones, including the diameter of the disc (mm), were measured using a Vernier caliper [41]. The antibacterial activity of green CMC/Ag-NPs was tested in-vitro, using the agar well diffusion method [42]. Overnight cultures of each strain, at 0.5 McFarland standard, were spread onto Luria-Bertani (LB) plates, which were pierced with a 6 mm diameter cork borer and loaded with 50 µL of CMC/Ag-NPs diluted in 1% DMSO at various concentrations (25, 50, and 100 μg/mL, *w*/*v*). After incubation, the radius of the inhibition zone was measured with a Vernier caliper. Minimal inhibitory concentrations (MICs) against tested organisms were determined using the microbroth dilution method and resazurin dye [43]. In 96 well plates, micro-dilutions of overnight developed culture strains (McFarland turbidity of 0.5) were cultivated in Luria–Bertani broth. CMC/Ag-NPs, at various concentrations (50, 25, 12.5, 6.25, 3.12, and 1.56 μg/mL), were added, and plates were incubated overnight at 37 °C. Any shift in culture color from blue dye to pink dye inside viable cells was visually examined [44]. The MIC value was determined by determining the lowest concentration of CMC/Ag-NPs, at which point the dye color changed. The MBC value was calculated when no colony appeared after plating directly into the agar plate. Antibacterial activity was described using the MIC index (MBC/MIC). Bactericidal activity is indicated by a MIC index of 1–2, whereas bacteriostatic activity is indicated by a MIC index of 4–16 [43].

### 2.5. Antifungal Activity

Many Fungal strains were used, such as *Candida albicans* ATCC 90028, *Aspergillus. terreus* (RCMB 02574), *A. niger* (RCMB 02724), and. *A. fumigatus* (RCMB 02568). These fungal strains were cultured on MEA plates and incubated for 5 days at 28 ± 2 °C, and then preserved at 4 °C for further use [18,45,46,47,48]. The agar diffusion test was performed according to Wayne [49], with minor modifications. Fungal strains were cultured separately on MEA plates and incubated at 28 ± 2 °C for 3–5 days. One milliliter of fungal spores solution (10^7^ spores/mL) was spread on agar MEA Plates. Then, 100 µL of each CMC-AgNPs, Ag, CMC, and nystatin were placed in wells (7 mm) and then incubated at 28 ± 2 °C. The diameter of the inhibitory zone was evaluated after 72 h of incubation.

In order to evaluate the inhibitory activity of each CMC-AgNP, Ag, CMC, and nystatin, MICs were determined via a broth microdilution method according to Sanguinetti et al. [50], with modifications proposed by Rojas et al. [51]. Initially, 1000μL of sterilized ME broth was added to the plate holes. Two-fold serial dilutions of each CMC-AgNP, Ag, CMC, and nystatin were performed to obtain a final concentration range from 0.5 to 0.008 mg/mL. An amount of 100 μL for each dilution was added. The microtiter plates, with 96 U wells, were incubated for 3 days at 30 °C. MICs of solutions were determined by visual reading of growth inhibition in the case of filamentous fungi.

### 2.6. Anticancer Activity

#### 2.6.1. Cell Lines

Human breast (MCF-7) and hepatocellular (HepG2) cancer cell lines (ATCC, Manassas, VA, USA) were cultured in DMEM with 10% FBS and 1% Pen/Strep solution (USA) in a 5% CO_2_ incubator at 37 °C.

#### 2.6.2. Cell Viability Assay

The MTT assay was used to determine cytotoxic activity [52,53]. The cells were seeded in 96-well plates and then allowed to grow for 24 h. After 24 h, those media containing various concentrations of CMC-AgNPs (100, 25, 6.3, 1.6 and 0.4 µg/mL) were replaced. After 48 h, 100 μL of MTT solution (5-mg/mL in PBS) was added to wells and kept for 4 h at 37 °C. Each well received a pipette containing 100 μL of DMSO to dissolve the formazan crystals. The plates were incubated for 10 min at 37 °C. At 570 nm, a microplate reader (Bio Tek, Winooski, VT, USA) assessed the optical density.

### 2.7. Assessment of Caspase-8 and -9 Activities and VEGFR-2

Caspase-8 (EIA-4863) and -9 (EIA-4860) were measured using an ELISA kit (DRG International Inc., Springfield, NJ, USA) for caspase-8 and -9 activities assessment. While VEGFR-2 was measured using an ELISA kit (Catalog #: ab213476) (Abcam, Cambridge, UK) following the manufacturer’s directions.

### 2.8. Flow Cytometric Analysis 

According to the manufacturer’s instructions, a cell cycle kit (Beckman Coulter) was used to assess the cell cycle in cultured cells using flow cytometry. While employing the recommended procedures, flow cytometry analysis (Beckman Coulter, Inc., Brea, CA, USA) was utilized to identify cell apoptosis in cultured cells using the Annexin V-FITC kit (BioVision, Milpitas, CA, USA). In brief, cells for all groups were cultured at 5 × 10^5^ cells/T75 flask and incubated overnight. After treatment with taxol (13.0 µg/mL) and CMC-AgNPs (7.9 µg/mL) or medium for 48 h, cells were allowed to grow in a 25 cm^3^ flask until they achieved 70–80% confluence. The cells were then rinsed in PBS and suspended at 5 × 10^3^–5 × 10^6^ cells/mL in 1 × binding buffer. Then, we added 100 μL of cell suspensions, 5 μL of dissolved PI, and 5 μL of annexin V-FITC solution, and incubated for 15 min in the dark. Following that, we added 400 μL of ice-cold 1 binding buffer and carefully mixed it. Flow cytometric analysis on a COULTER Flow Cytometer (Beckman Coulter) was used to identify apoptotic cells [54,55].

### 2.9. Statistical Analysis 

GraphPad Prism 8.0 (San Diego, CA, USA) analyzed all results. All results (three independent experiments) were shown as means ± standard deviation. The significance of the differences in the outcomes of all groups was examined using ANOVA and Tukey’s multiple comparisons tests. Statistical significance was defined as a *p* < 0.05.

## 3. Result and Discussion

### 3.1. Characterization of CMC-AgNPs

**Figure 1:**
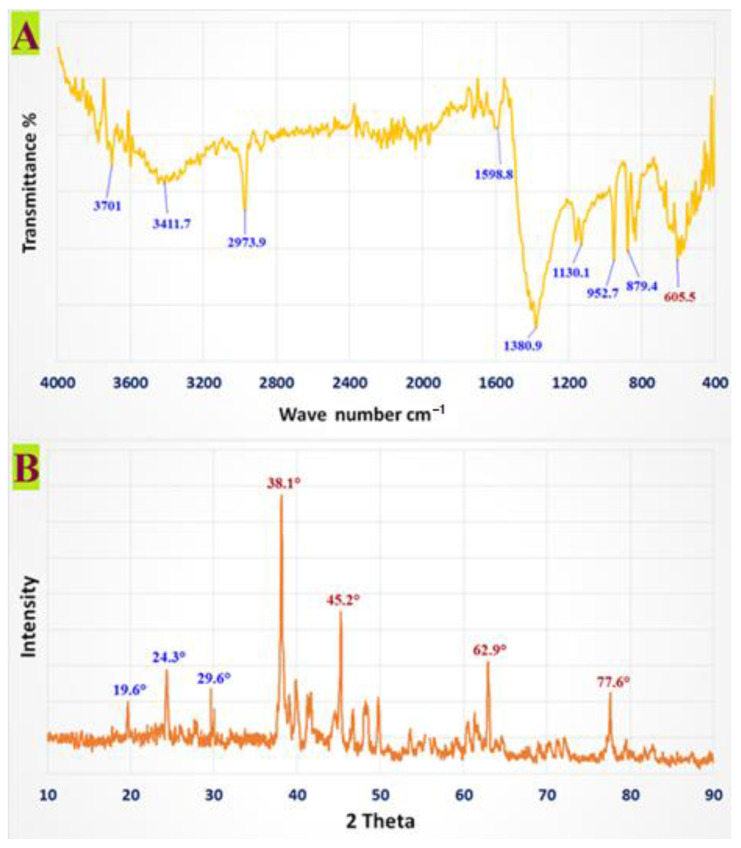
FTIR spectrum and XRD pattern of CMC-AgNPs.

The decrease in chemical interactions and stabilizing agents, which are crucial for nanoparticle formation and stability, was confirmed via the FTIR analysis of the CMC-AgNPs. This approach is frequently used to qualitatively examine nanostructures. The Ag NPs’ FTIR spectra showed characteristic absorption at 3701 cm^−1^, 3411.7 cm^−1^, 2973.9 cm^−1^, 1598.8 cm^−1^,1380.9 cm^−1^, 1130.1 cm^−1^, 948 cm^−1^, 879.4 cm^−1^, and 605.5 cm^−1^, which are associated with linkage groups (Figure 1A). Furthermore, the high peaks at 3701 cm^−1^ and 3411.7 cm^−1^ correlated to -OH group stretch vibrations. It is possible to assign both symmetric and asymmetric -CH_2_ to the band at 2973.9 cm^−1^. 

The band expansion of glucose generated a band at 1598.8 cm^−1^. The reduction of silver ions (Ag+) in CMC structures is complemented by hydroxyl group oxidation, as evidenced by a significant 1380.9 cm^−1^ signal for CMC-AgNPs. The peak shows C-O stretching vibration at 1130.1 cm^−1^. The absorption peaks at 948 and 879.4 cm^−1^ conform to the β-1,4—glucoside unit’s typical absorption. The presence of Ag in CMC-AgNPs resulted in a 605.5 cm^−1^ absorption peak. 

The X-ray diffraction pattern of AgNPs, stabilized with CMC, revealed several Bragg’s reflections with 2θ values of 38.1°, 45.2°, 62.9°, and 77.6°, corresponding to (111), (200), (220), and (311) sets of lattice planes of face-centered cubic (fcc) structures of metallic Ag, demonstrating that the synthesized silver nanoparticles were pure. The XRD data for the CMC-AgNPs were discovered to be quite close to JCPDSccard no. 04-0783. As a consequence, AgNPs were produced by the reduction of AgNO_3_ using CMC, as shown by XRD (Figure 1B), and indeed, the crystal structure supported earlier research [27]. The most notable diffraction peaks at 19.6°, 24.3°, and 29.6° further supported CMC’s crystalline structure.

**Figure 2:**
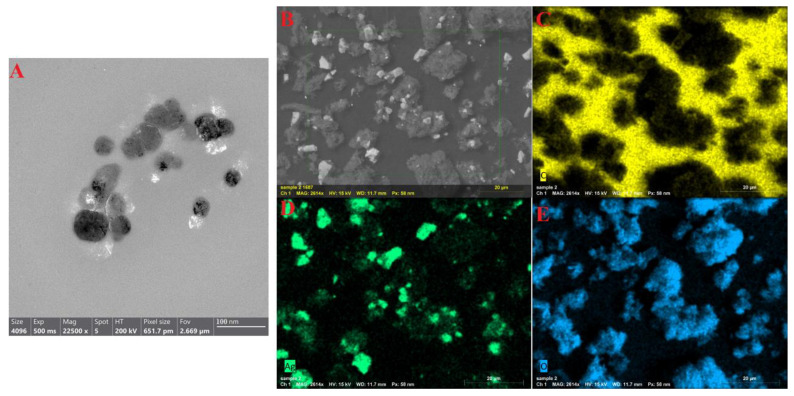
TEM image (**A**), SEM image (**B**), and SEM/EDX mapping analysis (**C**–**E**) of CMC-AgNPs.

The produced CMC-AgNPs were approximately spherical, poly-disperse, and ranged in size from 20 to 85 nm, according to the TEM image (Figure 2A). The particles were spherical and had an Ag core covered in a thin layer of CMC. Additionally, TEM images showed that the CMC layer around the Ag nanoform was uniformly dispersed. No aggregation was discovered when CMC-AgNPs were examined, confirming that the nanomaterials were totally coated in a polymer. SEM evaluation of the surface topography and crystalline size of CMC-AgNPs is depicted in Figure 2B. The shape of CMC-AgNPs was almost irregular. Nanostructures ranged in average particle sizes from 230 to 298 nm. The elemental composition of the CMC-AgNP powder was ascertained via EDX analysis. The EDX spectra of the CMC-AgNPs showed the presence of a number of clearly characterized elements linked to silver, oxygen, and carbon components in Figure 2C–E. There was carbon [C] and oxygen [O] in the mapping of the CMC, whereas the silver [Ag] map indicates the creation of Ag nanostructures.

### 3.2. Antibacterial Activity and MIC Index

Bacterial susceptibility to CMC-AgNPs was shown to differ depending on the strains used in the study. CMC-AgNPs, at a concentration of 100µg/mL, inhibited *Klebsiella oxytoca* & *Escherichia coli*, with a maximum inhibition zone of 18 ± 0.91 & 17 ± 0.63, respectively, while CMC-AgNPs at a concentration of 50 µg/mL inhibited *Klebsiella oxytoca* & *Escherichia coli*, with a maximum inhibition zone of 12 ± 0.34 & 12 ± 0.27, respectively, as shown in Table 1. The diameter of the inhibition zone for the CMC-AgNP sliver nanoparticles against *Staphylococcus aureus* & *Bacillus cereus* demonstrates that CMC-AgNPs exhibited less antibacterial activity compared with tested gram-negative bacteria. When both treatments were used at the same concentration, inhibition zones appeared, indicating that CMC-AgNPs had stronger antimicrobial properties than silver nanoparticles. The antibacterial effect of CMC-AgNPs could be due to an electrostatic interaction between positively charged silver ions and negatively charged microorganism cell membranes [44]. The MIC is known as the minimal effective dose of CMC-AgNPs necessary to prevent observable microbial growth within 24 h [56]. Using a resazurin-mediated microtiter plate test, we found that the greater diameter of inhibition exhibited with lower MIC values indicated strong antibacterial activity in bacterial strains (RMPA). Because the MBC/MIC ratio was more than 1, the MIC index values of CMC-AgNPs had a bactericidal effect against all tested bacterial strains, according to the findings. There was no significant difference in the effects of CMC-AgNPs on all pathogens. CMC-AgNPs displayed modest antibacterial activity against *Staphylococcus aureus* and *Bacillus cereus*, with MICs ranging from 25 to 50 µg/mL, while CMC-AgNPs had increased antibacterial activity against *Klebsiella oxytoca* and *Escherichia coli* with MICs value 12.5 µg/mL as shown in Table 2. The advantages of this assay over other antibacterial assays include increased sensitivity for tiny amounts of material, capacity to discriminate between bacteriostatic and bactericidal effects, and quantitative calculation of MIC are all benefits of this test over other antibacterial assays [57]. Therefore, the antimicrobial activity of the CMC@AgNPs solution was mainly attributed to the release of AgNPs, which could interact with cell membranes or penetrate into the cell interior [58].

Due to the synergism of antibacterial capability of AgNPs and CMC after combination, CMC-AgNPs were found to have enhanced antibacterial activity in the current study. The Ag-O coordination interactions between AgNPs and COO moieties of CMC, which increase the capacity of AgNPs to re-release Ag+ ions into the aqueous dispersion [59], may be the cause of the increased antibacterial activity observed. The precise mechanism behind the negligible antibacterial activity of CMCs has not been reported in prior investigations, similar to AgNPs. The hydroxyl groups of the gluco-pyranose monomers are present in CMC as well, which raises the possibility that it could react with both the bacterial cell wall and the cell membrane [60].

### 3.3. Antifungal Activity

**Figure 3:**
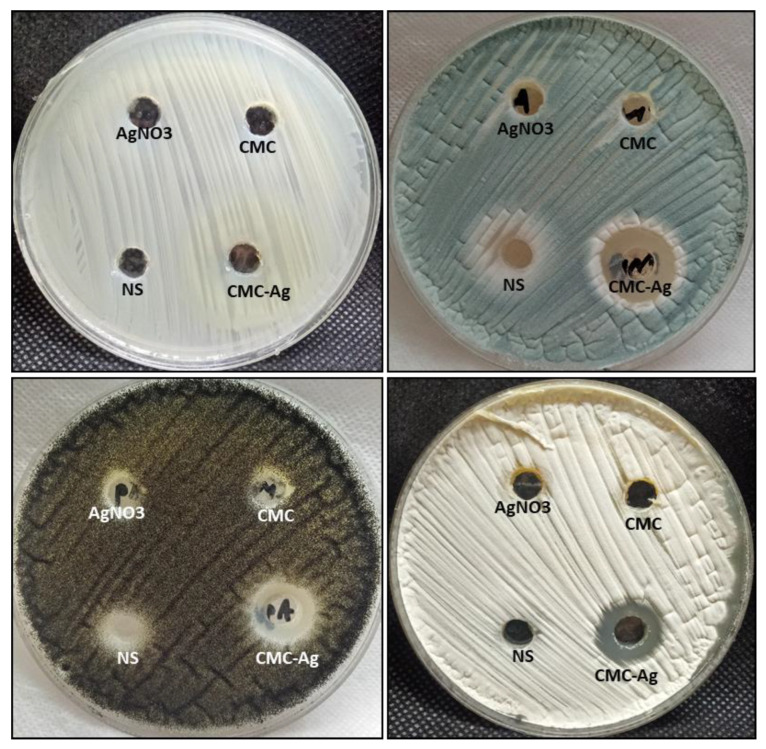
Antifungal activity of CMC-AgNPs and their start materials using agar well diffusion method.

In this study, the antifungal activity of CMC-AgNP nanocomposites was evaluated against unicellular and multicellular fungi, as illustrated in Figure 3. The results revealed that CMC-AgNPs have antifungal activity toward multicellular fungi only. The inhibition zone at a 0.5 mg/mL concentration was 19, 15, and 16 mm toward *A. fumigatus*, *A. nniger*, and *A. terreus*, respectively. Moreover, the MIC of CMC-AgNPs against *A. fumigatus*, *A. nniger*, and *A. terreus*, was 0.0312, 0.125, and 0.125 mg/mL, respectively (Table 3). Nystatin only had weak antifungal activity against *A. fumigatus* and *A. niger* but had no activity on *C. albicans* and *A. terreus*.

On the other hand, CMC-AgNPs had no activity on *C. albicans*, where it did not inhibit the growth of *C. albicans*. Additionally, both CMC and AgNO_3_ could not inhibit all tested fungal strains except *A. niger* only, where the inhibition zone was 9 and 8 mm, respectively. The rise in antimicrobial action is because of the stability of CMC-AgNPs in an aqueous medium since CMC keeps them from aggregating and increases the surface area of biomaterial compounds [61]. The mechanism of action of CMC-AgNPs is credited to the formation of bonds between AgNPs and COO− moieties of CMC; this leads to modifying the ability of NPs to produce and release Ag+ into aqueous dispersion, and consequently, the antimicrobial activity increases [62]. Silver ions disrupt biological membranes by invading metabolism mechanisms and damaging cell membranes by attaching to proteins and enzymes, which are key components of cells’ structural machinery, in particular to their R-SH groups [63].

### 3.4. Anticancer Activity

#### 3.4.1. Cytotoxic Effect of CMC-AgNPs against HepG2 and MCF-7 Cell Lines 

**Figure 4:**
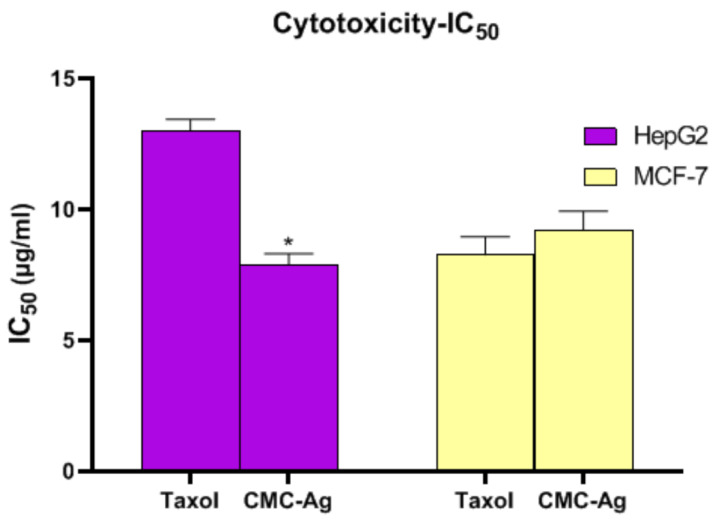
In-vitro cytotoxic activities of CMC-AgNPs against MCF-7 and HepG2 cell lines. The results are shown as the mean ± SD. * Significantly different from the Taxol group at *p* < 0.001.

The cytotoxic effect of CMC-AgNPs was examined for two cancer cell lines, HepG2 and MCF-7 cell lines for liver and breast cancer, correspondingly. As demonstrated in Figure 4, HepG2 cells presented lesser IC_50_ values (7.9 ± 0.41 µg/mL) than MCF-7 cells (9.2 ± 0.73 µg/mL), suggestive of the greatest cytotoxic effect of CMC-AgNPs on hepatic cancer cells. The IC_50_ for taxol was 13 ± 0.44 ug/mL for HepG2 cells and 8.26 ± 0.69 ug/mL for MCF-7 cells. 

Similarly, Saratale and his colleagues assessed the therapeutic potential of silver nanoparticles synthesized through a green route using Punica granatum leaf extract, which showed significant antioxidant and antidiabetic activities. Moreover, the HepG2 liver cancer cell line exhibited remarkable cytotoxic activity, assisting with the potential useful application of this nanomaterial in hepatic cancer therapy [64].

#### 3.4.2. Effect of CMC-AgNPs on VEGFR-2

**Figure 5:**
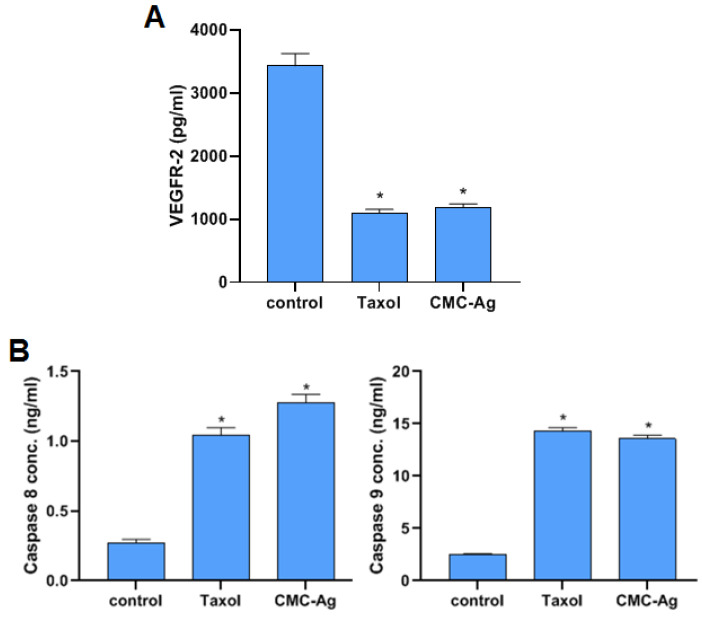
Effect of CMC-AgNPs on VEGFR-2 (pg/mL) in HepG2 cells compared to taxol (**A**); effect of CMC-AgNPs on caspase-8 and caspase-9 (ng/mL) in HepG2 cells compared to taxol (**B**). The results are shown as the mean ± SD. * Significantly different from the control (HepG2 cells) group at *p* < 0.0001.

To examine the effect of CMC-AgNPs on the angiogenic process, VEGFR-2 was assessed. As represented in Figure 5A, CMC-AgNPs significantly diminished the level of VEGFR-2 to 1187 ± 55.4 pg/mL, compared with the control (3429 ± 195 pg/mL; HepG2 cells). It is noteworthy that the effect of CMC-AgNP treatment on the VEGFR-2 levels was similar, with no statistical difference from the standard anticancer taxol (1097 ± 59.4 pg/mL). A key proangiogenic factor called VEGF is essential for the growth of the blood vessel network. It has been demonstrated that many different kinds of inorganic NPs prevent the growth of new blood vessels by preventing the VEGF-induced phosphorylation of VEGFR2, hence deactivating the downstream pathways. By inhibiting the VEGF/VEGFR2 pathway, silver NPs also had an antiangiogenic effect [65,66].

#### 3.4.3. Effect of CMC-AgNPs on Caspase 8 and 9 Activities

The influence of CMC-AgNPs on the apoptotic indicators caspase 8 and 9 is depicted in Figure 5B. The activity of caspase 8 and 9 was dramatically elevated with the treatment of HepG2 cells using CMC-AgNPs (1.274 ± 0.062 ng/mL and 13.57 ± 0.33 ng/mL, respectively) and with taxol (1.04 ± 0.058 ng/mL and 14.27 ± 0.35 ng/mL, respectively) in comparison to the control (0.273 ± 0.024 ng/mL and 2.503 ± 0.09 ng/mL, respectively). In agreement with this, a recent study conducted by Kumari et al. revealed that silver nanoparticles triggered the production of reactive oxygen species and stimulated apoptosis. Nanoparticles were shown to interact with caspase proteins such as caspase 9 through proline cysteine, glycine, and histidine amino acid residues [67]. Our findings that CMC-AgNPs triggered apoptosis in HepG2 cells through caspase-8 and -9-dependent protocols, are further supported by elevated caspase-8 and -9 activity.

#### 3.4.4. Effect of CMC-AgNPs on Apoptosis of HepG2 Cell Line

**Figure 6:**
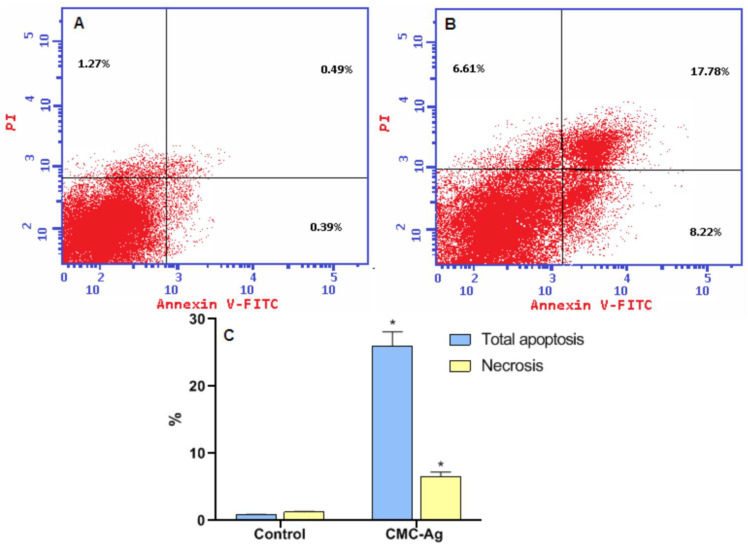
CMC-AgNPs induce apoptosis in the HepG2. (**A**) Control, (**B**) CMC-AgNPs, and (**C**) a graphical representation for % of apoptotic and necrotic cells. * Significantly different from the control group at *p* < 0.0001.

Figure 6 illustrates the distribution of cells in each quadrant according to necrosis, late apoptosis, live cells, and early apoptosis (Annexin V-positive cells). The percentage of total apoptosis for CMC-AgNPs treated cells was 26 ± 2.08% in comparison to the 0.88 ± 0.027% for the control. Moreover, for necrotic cells, the percentage significantly increased in CMC-AgNPs treatment (6.61 ± 0.609%). Control cells showed only 1.27 ± 0.09% necrosis. 

Silver nanoparticles possess unique cytotoxic features and can induce apoptosis and necrosis in various cancer cells, including breast, ovarian, lung, and cervical cancers [68,69,70]. Besides, a previous report indicated that green silver nanoparticles are inducers of apoptosis in the liver cells [70]. Accordingly, our results signify that the likely antitumor effect of these CMC-AgNPs is due to the induction of the apoptosis and necrosis of hepatic cancer cells.

### 3.5. Cell Cycle Analysis

**Figure 7:**
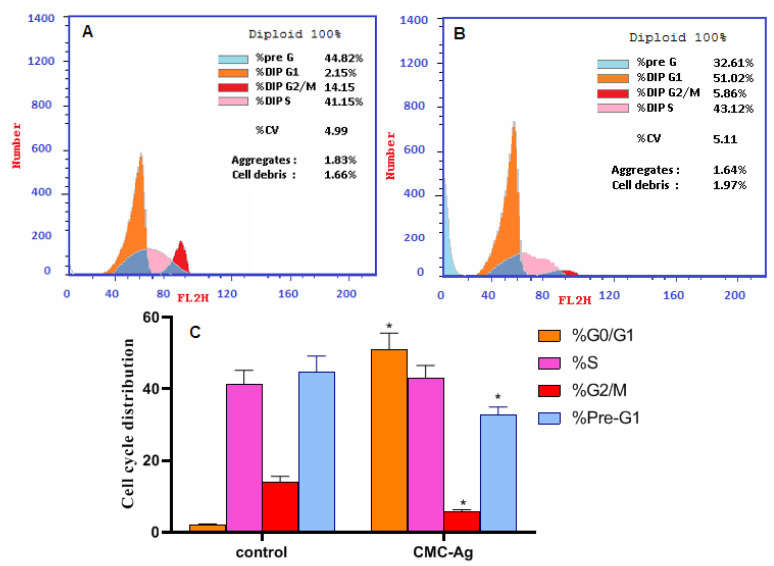
Flow cytometry analysis of the cell-cycle distribution of HepG2 cells. (**A**) Control, (**B**) CMC-AgNPs, and (**C**) a graphical represent of cell-cycle distribution analysis among different treated cells. * Significantly different from the control group at *p* < 0.0001.

The cell cycle of HepG2 cells is represented in Figure 7. Treatment with CMC-AgNPs led to a significant rise in apoptosis by increasing the number of cells in the G1/G0 phase of the cell cycle (51.02 ± 4.51%) compared to 2.15 ± 0.15% in control cells. Excitingly, treatment with CMC-AgNPs displayed a notable decrease in cells in the pre-G1 phase of the cell cycle (32.61 ± 2.34%) compared to 44.82 ± 4.35% in the control cells. CMC-AgNPs treatment, instead, resulted in a significant reduction in the number of cells accumulating in the G2/M phase of the cell cycle; an amount of 5.86 ± 0.43% versus 14.03 ± 1.52% for the control cells were present in this phase. However, CMC-AgNPs treatment produced no significant change in cells in the S phase (43.12 ± 3.41%) compared to the control cells (41.15 ± 3.99%). 

These findings show a shift in cell cycle dynamics in response to CMC-AgNPs treatment, indicating high efficacy. The increased number of cells in the G1/G0 phase helps the apoptotic shift. An arrest at the G1/G0 checkpoint determines their survival, with DNA damage that activates apoptosis-like programs [71]. In concert with our findings, it was formerly reported that glucose-capped silver nanoparticles influenced cervical cancer cells’ viability and cell cycle progression, suggesting their use alone or in combination with chemotherapeutics as a novel anti-proliferative treatment for cancer therapy [72]. 

## 4. Conclusions

This study synthesized CMC-AgNP nanocomposites through green and eco-friendly methods. Colloidal CMC-AgNPs were prepared by the reduction of Ag ions in the presence of CMC, giving CMC-AgNPs. Physiochemical characterizations were determined by FTIR, XRD, TEM, and SEM-EDX analysis. The antibacterial efficacy of this nanocomposite against Gram-negative and Gram-positive human pathogenic bacteria is promising. Likewise, it has antifungal activity against aspergilli, which causes aspergillosis disease. Furthermore, the anticancer impact of CMC-AgNPs is due to the induction of apoptosis and necrosis in hepatic cancer cells by increasing caspase-8 and -9 activity and decreasing VEGFR-2. CMC-AgNPs also arrested the cell cycle at the G1/G0 phase, according to cell cycle analysis. Herein, the prepared nanocomposite (CMC-AgNPs) shows promising results as an antimicrobial and anticancer agent, which can be recommended as an antimicrobial and anticancer drug after in-vivo studies.

## Figures and Tables

**Table 1:** Antimicrobial activity as indicated by a growth-inhibition zone of different concentrations of CMC-AgNPs and AgNPs against different strains of bacteria.

Bacterial Strains	Growth-Inhibition Zone in mm (Mean ± SD) as Caused by Different Concentrations of CMC-AgNPs and AgNPs				
	CMC-AgNPs 100 µg/mL	CMC-AgNPs 50 µg/mL	AgNPs 100 µg/mL	AgNPs 50 µg/mL	Ciprofloxacine
*Klebsiella oxytoca* ATCC 51983	18.0 ± 0.91	12.0 ± 0.34	13.0 ± 0.81	8.2 ± 0.51	13.0 ± 2.3
*Escherichia coli* ATCC 35218	17.0 ± 0.63	12.0 ± 0.27	14.0 ± 0.39	9.6 ± 0.51	15.0 ± 1.8
*Staphylococcus aureus* ATCC 25923	12.0 ± 0.41	8.5 ± 0.3	9.5 ± 0.37	7.5 ± 0.27	14 ± 3.2
*Bacillus cereus* ATTC 11778	11.0 ± 0.97	8.3 ± 0.6	8.5 ± 0.16	6.5 ± 0.37	16 ± 1.9

Each value is the mean ± SD of triplicate analysis.

**Table 2:** The MIC values were determined by colorimetric assay (resazurin), MBC (99.9% kill) and MIC/MBC ratio of CMC-AgNPs against *Klebsiella oxytoca*, *Escherichia coli*, *Staphylococcus aureus*, and *Bacillus cereus*.

Standard Bacterial Strains	MIC (μg/mL)	MBC (μg/mL)	MBC/MIC Ratio
*Klebsiella oxytoca* ATCC 51983	12.5	25.0	2
*Escherichia coli* ATCC 35218	12.5	25.0	2
*Staphylococcus aureus* ATCC 25923	25.0	100.0	4
*Bacillus cereus* ATTC 11778	50.0	150.0	3

**Table 3:** Inhibition zone and MIC of CMC-AgNPs compared to starting materials.

	*C. albicans*		*A. fumigatus*		*A. niger*		*A. terreus*	
	IZ/mm (0.5 mg/mL)	MIC mg/mL	IZ/mm (0.5 mg/mL)	MIC mg/mL	IZ/mm (0.5 mg/mL)	MIC mg/mL	IZ/mm (0.5 mg/mL)	MIC mg/mL
CMC	ND	ND	ND	ND	9	0.5	ND	ND
AgNO_3_	ND	ND	ND	ND	8	0.5	ND	ND
CMC-AgNPs	ND	ND	19	0.0312	15	0.125	16	0.125
NS	ND	ND	11	0.5	12	0.25	ND	ND

## Data Availability

The data used to support the findings of this study are available from the corresponding author upon request.
